# Learning Collaborative to Support Continuous Quality Improvement in Newborn Screening

**DOI:** 10.3390/ijns11030070

**Published:** 2025-08-27

**Authors:** Elizabeth Jones, Sikha Singh, Sarah McKasson, Ruthanne Sheller, Jelili Ojodu, Ashley Comer

**Affiliations:** Association of Public Health Laboratories, 7700 Wisconsin Avenue, Suite 1000, Bethesda, MD 20814, USA; elizabeth.jones@aphl.org (E.J.); sikha.singh@aphl.org (S.S.); sarah.mckasson@aphl.org (S.M.); ruthanne.sheller@aphl.org (R.S.); jelili.ojodu@aphl.org (J.O.)

**Keywords:** collaborative, continuous quality improvement, newborn screening, plan-do-study-act cycles, timeliness, association of public health laboratories (APHL), NewSTEPs

## Abstract

As newborn screening (NBS) programs deal with growing complexities, including adding new disorders to their screening panels, adopting new technologies/screening methods, and workforce shortages, there is a greater need for continuous quality improvement (CQI) to ensure the NBS system is meeting its primary goal of identifying infants with NBS disorders in a timely fashion. In 2019, the Health Resources and Services Administration’s (HRSA) Maternal and Child Health Bureau (MCHB) awarded funding to the Association of Public Health Laboratories’ (APHL) Newborn Screening Technical assistance and Evaluation Program (NewSTEPs) to address CQI in the NBS system through a collaborative, data-driven process. From 2019–2024, NewSTEPs funded 36 quality improvement (QI) projects from a variety of state NBS programs and research centers across the U.S., to address timeliness, detection of out-of-range results, communication of results, and/or confirmation of diagnosis. Thirty-three QI teams completed their projects, and 85% achieved their specified goal outlined in their aim statement. Despite limitations, the QI Projects Collaborative provided NBS programs with funding and resources to begin and sustain quality improvement initiatives. This model of a technical assistance and central resource center for CQI was effective in achieving quality improvements within the national NBS system.

## 1. Introduction

Newborn screening (NBS) is recognized as one of the most successful public health programs in the United States [1]. It is the practice of screening every newborn for harmful or potentially fatal disorders that are not apparent at birth. Early detection is critical to saving lives and preventing disability.

Although the Health and Human Services (HHS) Secretary’s Advisory Committee on Heritable Disorders in Newborns and Children (ACHDNC) has historically recommended certain disorders be included on the Recommended Uniform Screening Panel (RUSP), each state and jurisdiction determines the specific disorders for which it screens. As of April 2025, all states and jurisdictions universally screened for at least 31 of the 38 disorders on the RUSP [2]. Despite the RUSP, differences persist among state NBS panels. Variation among states and jurisdictions exists due to differences in state and territorial laws, the ability to increase NBS fees, as well as the availability of resources, infrastructure, and technology. In addition to incorporating new disorders into their panels, programs are committed to the ongoing enhancement of NBS processes. These initiatives are vital for ensuring quality practices across the NBS system.

The Newborn Screening Technical assistance and Evaluation Program (NewSTEPs), a program of the Association of Public Health Laboratories (APHL), is a national NBS resource center designed to increase harmonization of data, provide technical assistance and training to NBS programs, and assist states and jurisdictions with quality improvement initiatives. The NewSTEPs data repository, which began in May 2012 as a Cooperative Agreement (U22MC24078) from the Health Resources and Services Administration (HRSA), is a voluntary data resource center meant to ensure a uniform approach to data collection and information sharing [2]. NewSTEPs activities continue to be funded through HRSA as the NBS Excel program. Data elements collected within the repository include state and territorial demographics, disorders screened, NBS fees, health information technology elements, NBS state and territorial policies, demographic information for cases, case definitions, and quality indicators. As of April 2025, the data repository included self-reported data for all NBS disorders for 56 NBS programs, including all U.S. states, American Samoa, the Commonwealth of the Northern Mariana Islands, the District of Columbia, Guam, Puerto Rico, and the U.S. Virgin Islands [3].

NBS programs continue to deal with a growing number of complexities, including the pressure to add new disorders to their panels, adoption of new technologies and screening methods, as well as workforce and supply shortages [4]. Consequently, there is a growing need for continuous quality improvement (CQI) initiatives to ensure the NBS system is meeting its primary goal of providing intervention in a timely fashion to reduce the risk of disability and morbidity. In 2018, HRSA introduced a funding opportunity (HRSA-18-070) to address CQI in the NBS system, with an emphasis on timeliness activities, detection of out-of-range results, communication of results, and confirmation of diagnosis while relying on collaboration, quality improvement processes, and data to facilitate the process.

APHL was awarded this cooperative agreement (UG8MC31893) and launched the NewSTEPs Continuous Quality Improvement Program. As part of the NewSTEPs CQI Program, the Quality Improvement Projects Collaborative (QI Collaborative) began in 2019 to facilitate continuous improvement throughout the system. The goals of the collaborative were:

1. Assess needs and engage state and territorial NBS programs for participation in a multidisciplinary collaborative network focused on NBS quality improvement projects.
2. Coordinate and facilitate QI projects and data-driven outcome assessments, utilizing evidence-based QI methodologies within each participating state/jurisdiction NBS program.
3. Create a model for replication, sharing, and sustainability of NBS QI projects.

The learning collaborative approach has been used by other organizations, including HRSA and the Institute for Healthcare Improvement (IHI). The design of the learning collaborative was informed by the Institute for Healthcare Improvement’s Breakthrough Series model, which emphasizes structured peer learning, data-driven improvement, and iterative testing to drive system-level change [5]. The QI Collaborative also utilized the Model for Improvement (MFI) framework, an evidence-based model that provides a structured approach to improvement. This model focuses on understanding why a change is needed, identifying indicators of improvement, and determining potential changes that could lead to better outcomes. Plan-Do-Study-Act (PDSA) cycles were employed to test changes and evaluate the next steps to ensure the desired improvements [6]. CQI intends to create sustainable change by meeting the needs of partners, identifying a root cause, following a holistic approach, and empowering teams to make necessary changes [7,8].

This study aimed to evaluate the impact of establishing a national learning collaborative on both individual program-level quality improvement efforts and broader improvements in newborn screening quality indicators.

## 2. Materials and Methods

### 2.1. Collaborative Formation and Criteria for Participant Inclusion

APHL sought applicants for the QI Collaborative through a Request for Proposal (RFP) process, where newborn screening programs could request funding to participate in a self-identified NBS CQI project. APHL received applications from a variety of NBS programs and other agencies on a range of topics focused on test method improvement, transit strategies, interoperability, specimen quality, and follow-up processes. APHL also offered supplemental funding for QI projects focused on continuity of operations planning (COOP) activities. Barriers identified through a needs assessment and the RFP process that the projects attempted to address are shown in Table 1.

Applications for the Collaborative were solicited in four funding cohorts, with subject matter experts conducting evaluations based on the following criteria:

1. Project Data- well-defined problem and plans for collecting baseline data and measuring data
2. Project Charter/Action Plan- change ideas specified, project team members representing the NBS system identified with project roles defined, and demonstration of staff and leadership support
3. Budget/Justification- funding needs and how they relate to the goals and objectives of the project
4. Impact- why the project is important, how it will improve the NBS system/community served
5. Sustainability- how activities will be sustained upon completion of the funding cycle

**Figure 1:**
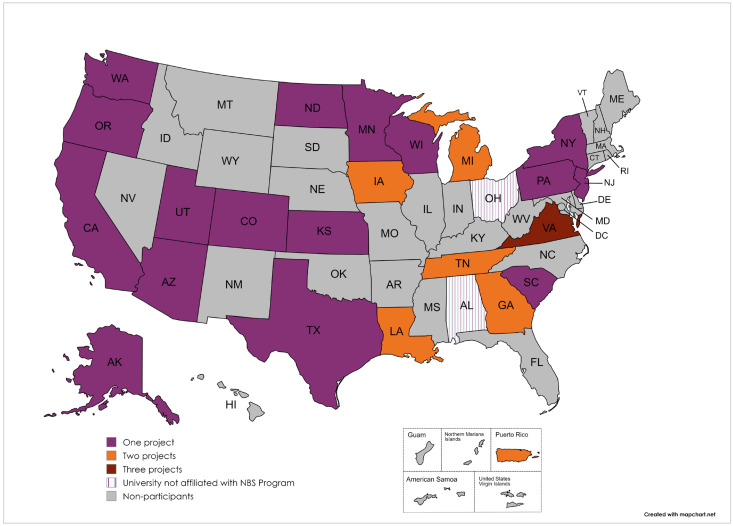
Funded states/jurisdictions that completed QI projects.

APHL approved funding for 36 QI projects from 28 agencies. Three awardees opted to end the agreement before completion of the proposed project due to competing priorities and external conflicts, for a total of 33 CQI projects completed (N = 33). Among the 25 agencies that completed their QI projects, 23 were from state and jurisdictional public health agencies, and two were from university centers. Several agencies applied for and completed more than one project (Figure 1).

### 2.2. QI Collaborative Engagement and Activities

In 2020, the NewSTEPs CQI Program established the Quality Improvement Subcommittee to guide the QI Collaborative and engage the community. This group also aimed to expand the culture of continuous quality improvement (CQI) within NBS. Their expertise was instrumental in shaping the trainings and opportunities offered for the QI Collaborative.

The QI Collaborative lasted from June 2019 to August 2024 and graduated four cohorts. Awardees were funded for varied lengths of time, between one and five years (with the final year being a no-cost extension period). QI projects focused on at least one of the following major topic areas: timeliness, identification and follow-up of out-of-range results, communicating NBS results, and confirming diagnosis. Individual teams included a variety of individuals within the NBS system, including laboratory, follow-up, and data/informatics staff.

QI Collaborative participants had access to a variety of resources, training, and technical assistance. Teams were required to join monthly virtual meetings with project coaches, attend training webinars, and participate in annual national CQI meetings. Optional bi-monthly discussion groups were also hosted for participants. Additionally, the NewSTEPs CQI Program offered other trainings, including a Greenbelt Lean Six Sigma Course.

Each QI project was assigned to a project coach who met monthly with the team to discuss project goals, PDSA cycles, challenges, successes, and strategies for measuring outcomes. The participants also submitted monthly reports, quarterly action plans, and annual and final reports to ensure the success of their projects.

Bimonthly discussions were held to foster innovation, problem solve, and network. The NewSTEPs CQI Program offered 20 QI webinars on topics ranging from Introduction to Measuring and Interpreting Quality Indicator Data and Tools for Tracking and Measuring Improvement (see Table A1 in Appendix A). Further, a six-part webinar series highlighted fifteen QI projects from the Collaborative to support a community of CQI.

The NewSTEPs CQI Program hosted a three-part virtual national CQI meeting in 2021, as well as two in-person meetings in August 2022 and April 2023. The format of the meetings supported learning new QI strategies as well as sharing NBS improvements and experiences across projects. The national meetings included topics such as CQI Principles for Sustaining Improvement, Tools for Tracking and Improving NBS, and Championing a CQI Culture.

Several mechanisms were used to communicate with QI Project participants and encourage them to collaborate, including a NewSTEPs CQI newsletter, a QI Projects Collaborative lead directory, and an NBS CQI-specific listserv hosted on APHL’s colLABorate community. Additionally, the NewSTEPs CQI website evolved to include information about CQI and its value, archived webinars, and tools on how to initiate a QI project.

### 2.3. Evaluation Criteria for QI Collaborative

Improvement was evaluated for individual QI projects and for the entire QI Collaborative. Individual evaluation for QI projects was assessed by reviewing run charts/control charts submitted by teams via monthly and final reports. Improvement was defined as meeting the goal outlined in the aim statement with evidence in a run chart by the completion of the project.

Success for the QI Collaborative as a whole was evaluated by using annual quality indicator data that teams were required to submit to the NewSTEPs Repository via webform or CSV uploads. The quality indicators are guided by detailed definitions to promote standardization [9]. NewSTEPs validates data by performing targeted outreach as needed. The analysis included baseline data from 2018 before the Collaborative began, to 2022 when data was most complete, accounting for the lag time for entering data into the data repository. The focus for analysis was on priority quality indicators (Table 2), including the percentage of unsatisfactory dried blood spot (DBS) specimens, the percentage of specimens missing essential information and timeliness (e.g., time of collection, transit time from specimen collection to receipt at screening laboratory and time elapsed from birth to reporting out results) [10]. Descriptive statistics were used to summarize quality indicator data and comparisons of medians between participating and non-participating QI Collaborative programs. Standard statistical tests were not applied due to heterogeneity of interventions and varying sample sizes.

## 3. Results

### 3.1. Quality Improvement Metrics

Most QI projects focused on timeliness (*n* = 20, 61%), followed by communication of NBS results (*n* = 6, 18%). A smaller number of QI projects addressed identification of out-of-range results (*n* = 3, 9%), confirming diagnosis (*n* = 2, 6%), and emerging issues such as continuity of operations planning (*n* = 2, 6%). Eighty-five percent (*n* = 28) of QI projects made improvements as identified in their aim statement. Fifteen percent (*n* = 5) of QI projects made improvements for some months; however, they did not maintain substantive improvement by the end of their project period with the metric outlined in their aim statement or remained stagnant with their progress (Table 3). All six teams that were in the Collaborative for the entire five-year period met their goals as detailed in their aim statement.

**Figure 2:**
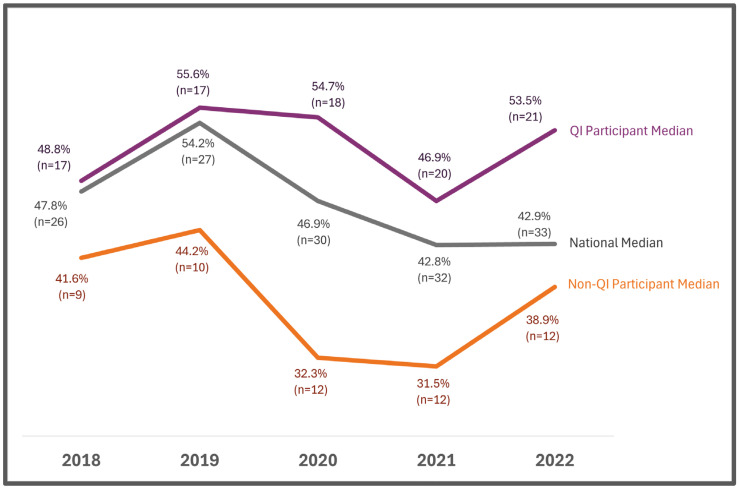
Reporting time-critical results to a medical professional (% Specimens) within five days of life: QI projects collaborative vs. non-participants and national median.

**Figure 3:**
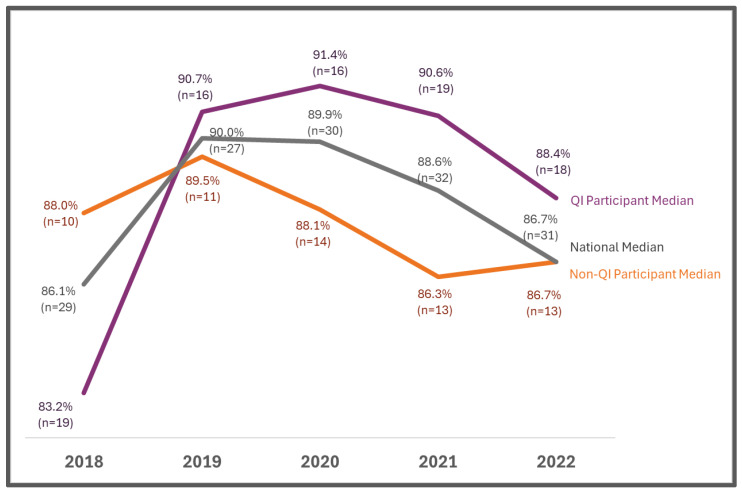
Reporting all results to a medical professional within seven days of life (% specimens): QI Projects Collaborative vs. non-participants and national median.

In 2022, the median percentage of specimens with time-critical results reported out within five days of life was higher for programs participating in the QI Collaborative compared to non-participating programs (53.5% vs. 38.9%) and higher than the national median (42.9%). Additionally, after 2018, there was an upward trend for this metric for QI Collaborative participating programs, and the inverse was true for non-participating programs (Figure 2). In 2022, the median percentage of all specimens reported out within seven days of life was higher for QI Collaborative participating programs compared with non-participating programs (88.4% vs. 86.7%) and higher than the national median (86.7%). Additionally, after 2018, there was an upward trend for this metric for QI Collaborative participating programs, and the inverse was true for non-QI Collaborative participating programs (Figure 3).

**Figure 4:**
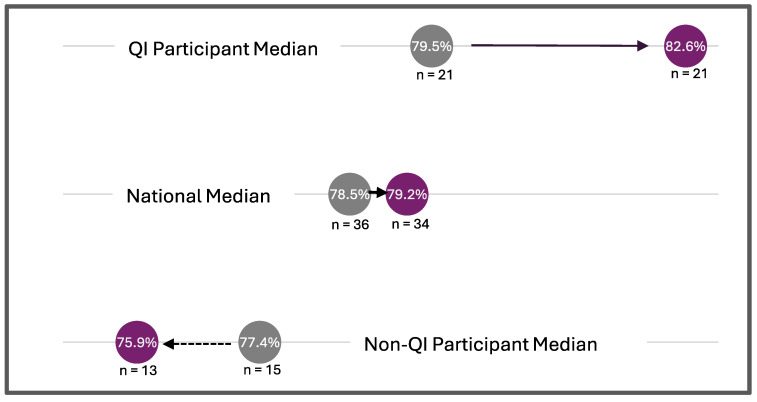
Change in the median percent of specimens received at the laboratory within two days from collection between 2018 and 2022. Each group’s 2018 median value is represented by a gray circle, and the 2022 median value by a purple dot. Lines connecting the two points illustrate the direction of change over time.

**Figure 5:**
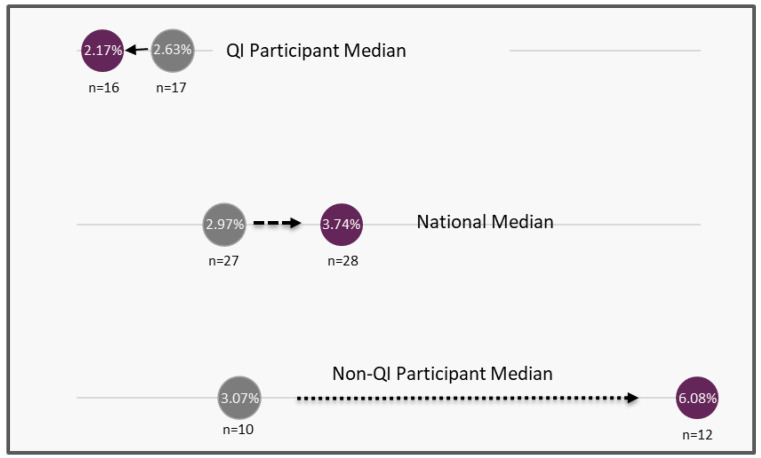
Change in the median percent of specimens with missing demographics between 2018 and 2022. Each group’s 2018 median value is represented by a gray circle, and the 2022 median value by a purple dot. Lines connecting the two points illustrate the direction of change over time.

Baseline data before the Collaborative (2018) and the latest, most complete dataset (2022) were compared. The median percentage of specimens received at the screening laboratory within two days from collection increased for the QI participants (79.5% to 82.6%) and the national median increased as well (78.5% to 79.2%) between 2018 and 2022, while it decreased for those not in the collaborative (77.4% to 75.9%) (Figure 4). The median percentage of specimens with missing demographics decreased for the QI Collaborative group (2.63% to 2.17%) between 2018 and 2022, while the national median (2.97% to 3.74%) and the non-Collaborative group median (3.07% to 6.08%) increased (Figure 5).

### 3.2. Program Evaluation

Project teams were required to complete an annual report at the end of each project year as well as a final report at the end of their project to gather feedback on team collaboration, relationships with other teams and partners, confidence with quality improvement tools, engagement in the QI Collaborative and usefulness of the resources provided by the NewSTEPs CQI Program. Although there were 33 project teams involved in the collaborative, 31 team evaluations were included in the analysis. Two project team evaluations were excluded because multiple projects were conducted within the same state, and the state submitted one evaluation for both projects. Since the project teams, while sharing a principal investigator, were composed of different members, we chose not to assume that the submitted responses were representative of both teams.

Of the 31 responses, 94% (*n* = 30) of teams agreed that they sought out and applied evidence-based practices to guide the work of their QI project(s). Ninety-seven percent (*n* = 31) of teams agreed that their team fostered a culture of continuously improving communication. By the end of the QI Collaborative, most teams were confident in the use of the QI tools, particularly PDSA cycles (*n* = 31, 97%), action plans (*n* = 31, 100%), aim statements (*n* = 31, 97%), run charts (*n* = 27, 87%) and quality measures (*n* = 29, 93%). Ten percent (*n* = 3) of teams were not confident in the use of control charts, and 13% (*n* = 4) were not confident in change packages.

All 31 project teams stated that they always or occasionally applied the following skills they learned to their project: communication, CQI, team-based relationships, and evidence practices. While 85% (*n* = 25) of project teams always or occasionally applied community-based collaborations.

The teams found the resources offered by the NewSTEPs CQI Program, including coaching calls, monthly reports, action plans, webinars, and national meetings, as either somewhat or extremely helpful. Continued access to CQI refresher courses (*n* = 28, 90%), an online subscription to CQI resources (*n* = 26, 84%), subject matter experts (*n* = 22, 71%), QI coaches (*n* = 18, 58%) and publication support (*n* = 17, 55%) were the most cited helpful resources in the 31 program evaluation questions. They also stated they wanted more opportunities for cross-cutting collaborations with other teams and resources for community engagement.

## 4. Discussion

This study set out to examine whether participation in a national learning collaborative—grounded in CQI principles—was associated with measurable improvements in newborn screening quality indicators, thereby contributing to system-level advancement across programs.

### 4.1. Key Findings

The evaluation of the QI Collaborative revealed several key findings that underscore the effectiveness of the initiative. Notably, ninety-four percent of teams sought out and applied evidence-based practices, and ninety-seven percent fostered a culture of continuously improving communication. Additionally, the high confidence in using QI tools, such as PDSA cycles, action plans, aim statements, run charts, and quality measures, suggests that the training and resources provided by the NewSTEPs CQI Program were effective in enhancing participants’ skills. The positive feedback on resources such as coaching calls, webinars, and national meetings indicates that these elements are crucial for supporting teams in their QI efforts. However, the lower confidence in using control charts and change packages highlights areas where further training and support may be needed.

From 2018 to 2022, there was improvement for QI Collaborative participating programs on many quality indicators, including changes in the percentage of missing demographics, percent of specimens received within two days of collection, time-critical results reported within five days of life, and all results reported within seven days of life. Additionally, QI Collaborative participating programs performed better than non-QI Collaborative participating programs and the national median for these quality indicators (Figure 2, Figure 3, Figure 4 and Figure 5).

### 4.2. Strengths

One of the strengths of this evaluation is the comprehensive nature of the data collection, including annual and final reports from all project teams. This approach provided a robust dataset for analysis.

The participation of 23 newborn screening programs and two university centers enabled broad dissemination of CQI technical assistance and peer learning, amplifying the collaborative’s reach and potential impact on national system improvements.

### 4.3. Challenges

The COVID-19 pandemic posed significant challenges for many NBS programs. Public health laboratories and healthcare centers’ ability to respond to new project requests and needs was limited and delayed. The delays and workforce impacts of the pandemic may have been a major contributor to the loss of some early improvement gains seen from the collaborative in 2021.

Additionally, projects that involved external factors (e.g., timeliness-transit projects with couriers, education to parents) outside of the program’s control reported more challenges. Competing priorities, such as adding new disorders to NBS panels, updating laboratory information management systems, and revising bloodspot cards, may have affected the program’s ability to dedicate time and effort to the QI project and are not fully captured in the results.

When evaluating, the variation in specificity of the customized aim statements for each QI project made it difficult to make comparisons across the Collaborative. Some QI projects focused on one process or outcome measure, while others included several. Furthermore, not all aim statements were tied to the NewSTEPs Quality Indicators, making it challenging to evaluate the Collaborative using these metrics.

### 4.4. Limitations

There was potentially some self-selection bias among participants in the QI Collaborative. For instance, it is possible that states that joined the QI Collaborative had more resources and engagement in QI activities to begin with, thus confounding their improvement. Non-Collaborative participants could have also implemented quality improvement projects during this same time frame outside of the Collaborative, which also limits analysis of the impact of the Collaborative.

Submitting quality indicator data to the NewSTEPs Repository was a requirement; however, the ability to submit quality indicators varies from program to program due to challenges (i.e., staff time, expertise, challenges with LIMs, etc.). Therefore, complete data sets were not available across all years and all programs. Further analysis could be done to measure this impact, if any. Data submitted to the NewSTEPs Repository is self-reported by the NBS programs. Awardees not associated with an NBS program were not required to submit quality indicator data since they did not have access to the information.

Additionally, some QI projects may not have shown progress with a specific metric; however, their programs may have already been performing above the national median/benchmark for quality indicators. Conversely, other QI projects that saw improvement may have started below the national median/benchmark.

Analysis of the participants’ overall evaluation of the experience participating also has some limitations to consider, such as potential biases in self-reported data and the scope of the evaluation, which may not capture all aspects of the QI Collaborative’s impact. Furthermore, since the annual and final reports were completed as a team, the individual experiences of team members may have varied, and these differences were not captured in the evaluation.

### 4.5. Impact

Although not all teams achieved the specific outcomes outlined in their original aim statements, this does not diminish the meaningful progress made throughout the project. CQI is as much about learning and adapting as it is about reaching improvement goals. Through the CQI process, teams discovered that some aim statements may not have been ideally suited to their context, or that the initial metrics selected did not effectively capture the changes they were striving to measure.

Importantly, teams used CQI methods to test small-scale changes before considering broader implementation. This approach allowed for thoughtful experimentation, learning, and refinement. Even if large-scale improvements were not fully realized during the project period, every team gained valuable insights into their systems and processes.

These lessons have laid a stronger foundation for ongoing improvement efforts and have equipped teams with the tools and knowledge to continue driving positive change beyond the scope of this initiative. The success of this learning collaborative demonstrates the value of structured, CQI-based initiatives in driving measurable improvements in newborn screening systems and supports the continued investment in similar national efforts to foster shared learning, technical capacity, and system-wide advancement.

## 5. Conclusions

The NewSTEPs CQI Program led the Continuous Quality Improvement Initiative from 2019 to 2024, which funded the completion of 33 QI projects and four cohorts across the U.S. The projects focused on addressing key issues in the NBS system, including timeliness, identification of out-of-range results, confirming diagnosis, communicating results, and emerging issues. The QI Collaborative provided NBS programs with the necessary funding, resources, and support needed to begin and sustain quality improvement initiatives.

Although the COVID-19 pandemic posed challenges, many of the QI projects succeeded in improving key quality metrics. Additionally, NewSTEPs data extracted in October 2024 revealed that Collaborative participating programs outperformed non-Collaborative participating programs and exceeded the national median in 2022 on many metrics including missing demographics, percent of specimens received (within two days of collection), percent of specimens with time-critical results reported out (within five days) and percent specimens with all results reported out (within seven days).

NBS programs received a strong foundation in CQI principles and tools that they can apply across their program. Collaborative participants shared their projects during national webinars, poster presentations, and national conferences to continue the spread of knowledge learned and improvements gained. The success of this initiative reinforces findings from other sectors showing that sustained investment in structured learning collaboratives can drive meaningful improvements, foster innovation, and build long-term capacity across systems [11].

The learning collaborative proved to be instrumental in supporting quality improvement (QI) in NBS processes. However, each program had an individual goal, as a collaborative, improvements to timeliness, processes for identifying and following up on out-of-range results, removing barriers for communicating NBS results, and/or confirming diagnoses were made. This model supported CQI learning and application to newborn screening-specific needs.

## Figures and Tables

**Table 1:** Barriers addressed by the QI Collaborative.

Project Category	Barriers
Timeliness	Lack of awareness at birthing centers/hospitals and midwives for timely specimen collection and transport Frequent staff turnover at birthing centers/hospitals Timing of courier pick-ups Lack of courier service on weekends/holidays Lack of resources (staff, equipment, funds) Lack of weekend and holiday laboratory testing Manual data entry of specimens and demographic info Lack of health information technology (HIT) systems for electronic messaging
Identification and Follow-up of Out-of-Range Results	Rushed addition of disorder(s) and validation of assay and setting cutoffs Limited staff and expertise Lack of feedback from clinician/follow-up on NBS results Inability to adopt new methods of detection/technology
Communication of NBS results to providers/parents	Lack of awareness or urgency from parents Lost to follow-up of cases due to incorrect or missing information on dried blood spot (DBS) card Providers do not understand the next steps when they are told about an out-of-range results
Diagnosis Confirmation	Poor communication with specialists Limitations with interoperability or data systems Delayed notification/no feedback loop to NBS programs
Emerging Issues	NBS programs do not have or have not practiced continuity of operations plans (COOP)

**Table 2:** Priority Quality Indicators used for collaborative evaluation.

Quality Indicator [10]	Subcategory
Unsatisfactory Specimens	a. Percent of unacceptable dried blood spot specimens due to improper collection
Unsatisfactory Specimens	b. Percent of unacceptable dried blood spot specimens due to improper transport
Missing Essential Information	Percent of dried blood spot specimens with at least one missing state-defined essential data field upon receipt at the laboratory
Unscreened Newborns	Percent of newborns without a valid dried blood spot newborn screen
Lost To Follow-Up	Percent of infants that have no recorded final resolution with the newborn screening program by 12 months of age following the receipt of an unacceptable dried blood spot specimen
Lost To Follow-Up	b. Percent of infants that have no recorded final resolution with the newborn screening program by 12 months of age following a borderline result for which a subsequent dried blood spot specimen was requested for repeat screening
Lost To Follow-Up	c. Percent of infants that have no recorded final resolution with the newborn screening program, by 12 months of age, following an out-of-range result from a dried blood spot screen requiring clinical diagnostic workup by an appropriate medical professional
Timeliness	Proportion of first dried blood specimens collected in the specified time categories in units of hours from birth.
Timeliness	b. Proportion of first dried blood spot specimens received at the designated newborn screening laboratory in the specified time categories in units of days from specimen collection
Timeliness	c. Proportion of first dried blood spot specimens with normal or out-of-range results for any disorder reported to a medical provider in the specified time categories in units of days from specimen receipt at the designated newborn screening laboratory.
Timeliness	d. Proportion of first dried blood specimens with normal or out-of-range results for any disorder reported to a medical provider in the specified time categories in units of days from birth.

**Table 3:** QI collaborative and results.

State Project Team	Years Involved	Project Type	Result As It Relates to Goal
Arizona	2020–2021	Timeliness	Unsatisfactory specimen rate improved
California	2020–2023	Timeliness	Transit time improved
Colorado	2019–2021	Timeliness	Did not reach goal
Georgia 2	2019–2021	Timeliness	Unsatisfactory specimen rate improved
Kansas	2020–2023	Timeliness	Transit time improved
Louisiana 1	2019–2024	Timeliness	Time to result reporting improved
Louisiana 2	2020–2021	Timeliness	QI model implemented
New Jersey	2021–2023	Timeliness	Transit time improved for pilot participants
New York	2019–2024	Timeliness	Transit time improved
Oregon	2022	Timeliness	Transit time improved
Pennsylvania	2020–2021	Timeliness	Time to result reporting improved
Puerto Rico 1	2019–2024	Timeliness	Time to result reporting improved
Puerto Rico 2	2019–2024	Timeliness	Time to result reporting improved
South Carolina	2019–2024	Timeliness	Transit time improved
Tennessee 1	2019–2022	Timeliness	Time to result reporting improved
Texas	2021–2024	Timeliness	Did not reach goal
Virginia 1	2019–2024	Timeliness	Time to result reporting improved and decreased missing essential data
Virginia 2	2020–2023	Timeliness	Time to result reporting improved
Washington	2021–2022	Timeliness	Transit time improved
Wisconsin	2020–2021	Timeliness	Time to result reporting improved
Georgia 1	2019–2021	Identification of out-of-range results	Positive predictive value for specific disorders improved
Tennessee 2	2021–2022	Identification of out-of-range results	Positive predictive value for specific disorders improved
Utah	2021–2024	Identification of out-of-range results	Did not reach goal
Iowa 1	2019–2022	Communication of results	Did not reach goal
Michigan 1	2021–2022	Communication of results	Parental awareness of NBS improved
Michigan 2	2022–2023	Communication of results	Midwife reporting rates improved
Minnesota	2019–2023	Communication of results	Long-term follow-up improved
North Dakota	2020–2022	Communication of results	Long-term follow-up improved
Univ of Alabama at Birmingham	2021–2024	Communication of results	Documented Sickle Cell Disorder follow-up practices across states
Alaska	2019–2021	Confirming diagnosis	Critical Congenital Heart Disease detection rates determined
Cincinnati Children’s	2021–2024	Confirming diagnosis	Did not reach goal
Iowa 2	2021–2022	Emerging issues	Developed an enhanced continuity of operations plan
Virginia 3	2021–2023	Emerging issues	Developed an enhanced continuity of operations plan

## Data Availability

The data presented in this study are available on request from the corresponding author. The data are not publicly available due to data security and privacy outlined in memoranda of understanding between APHL and state public health laboratories.

## Appendix

**Table A1:** Quality Improvement (QI) Webinars Offered.

Webinar Topic	Date	Description
NewSTEPs QI Projects: Awardee Kickoff Call	November 2019	Introduction and overview of the QI Projects Collaborative (Cohort 1)
NewSTEPs QI Projects: Cohort 1 Webinar	January 2020	Tools and strategies for starting a continuous quality improvement project
NewSTEPs QI Projects: Cohort 2 Kick-off Call	April 2020	Introduction and overview of the QI Projects Collaborative (Cohort 2)
NewSTEPs QI Projects: Teach and Learn, Steel Shamelessly, Share Seamlessly	June 2020	Introduction to relational coordination and strategies for building collaborative relationships to achieve project goals
NewSTEPs CQI National Webinar: So, You Want to Start a Quality Improvement Project	July 2020	Strategies for identifying a strong aim statement, ways to gain organizational buy-in, and techniques for testing change and measuring impact
NewSTEPs QI Projects: Introduction to Measuring and Interpreting Quality Improvement Data: Introduction to Run Charts	September 2020	Introduction to run charts and tracking improvement
NewSTEPs CQI National Webinar: Tools for Tracking and Measuring Improvement	October 2020	Introduction to a suite of tools templates, and software for designing charts and diagrams useful for tracking and measuring improvement including process maps, run charts, and control charts
NewSTEPs QI Projects: Introduction to Measuring and Interpreting QI Data: Run Charts, a Deeper Dive	November 2020	Introduction to building and interpreting run charts
NewSTEPs QI Projects: Improvement Burnout and the Power of Small Wins	January 2021	Introduction to quality improvement burnout and how to avoid it by taking the time to celebrate small wins
NewSTEPs QI Projects: Celebrating Successes and Strategies for Holding the Gains	March 2021	A continuation of the celebration of small wins and strategies for sustaining project successes
NewSTEPs QI Projects: Cohort 3 Kick-off Call	April 2021	Introduction and overview of the QI Projects Collaborative (Cohort 3)
NewSTEPs QI Projects: Introduction to the Change Package	June 2021	Strategies for gaining or increasing buy-in to advance improvements within quality improvement projects
NewSTEPs QI Projects: Getting Buy-In to Advance Improvement	October 2021	Strategies for gaining or increasing buy-in to advance improvements within quality improvement projects
NewSTEPs QI Projects: Cohort 4 Kick-Off Call	February 2021	Introduction and overview of the QI Projects Collaborative (Cohort 4)
NewSTEPs QI Projects: Tools for Every Stage of the PDSA	April 2022	Outline specific tools available at each stage of the Plan-Do-Study-Act to assist teams when performing rapid cycle testing and PDSA tracking strategies
NewSTEPs QI Projects: Strategies for Defining the Problem	June 2022	Ideas to systematically define a problem and identify a possible change idea
NewSTEPs QI Projects: Sustaining Momentum	September 2022	Approaches to maintain impetus as QI project continues; NewSTEPs repository visualizations utility in QI work shared
NewSTEPs QI Projects: NBS Collection Device Modification	December 2022	Demonstration of how to use quality improvement tools when changing the newborn screening collection device and what quality control measures to monitor
Variation Knowledge: Understanding and Managing Variation to Improve Outcomes	March 2023	Review independent system fundamentals crucial to improving the performance of systems; distinguish between variation types to respond to improvement
